# Supplementary material for: Excitonic-Vibrational Interaction at 2D Material/Organic Molecule Interfaces Studied by Time-Resolved Sum Frequency Generation
Source: Nanomaterials (Basel). 2024 Nov 25;14(23):1892. doi: 10.3390/nano14231892 (PMC11643653; doi:10.3390/nano14231892)
Supplement: Supplementary file 1 [file nanomaterials-14-01892-s001.zip › nanomaterials-3294576-supplementary.pdf]

Supplementary Materials

Excitonic-Vibronic Interaction at 2D Material/Organic Molecule Interfaces Studied by Time-Resolved Sum Frequency Generation

Huiling Chen [1], Yu Lian [1], Tao Zhou [1], Hui Li [1], Jiashi Li [1], Xinyi Liu [1], Yuan Huang [2] and Wei-Tao Liu [1*]

[1] Physics Department, State Key Laboratory of Surface Physics, Key Laboratory of Micro and Nano Photonic Structures [Ministry of Education (MOE)], Fudan University, Shanghai 200433, China

[2] School of Integrated Circuits and Electronics, MIIT Key Laboratory for Low-Dimensional Quantum Structure and Devices, Beijing Institute of Technology, Beijing 100081, China

* Correspondence: wtliu@fudan.edu.cn

I. Fitting parameters of spectra in the main text

Table S1. Fitting parameters for the static sum-frequency (SF) spectra (for Fig. 3 in the main text)

**MoS_2/PMMA**			**WS_2/PMMA**			**Silica/PMMA**		
ω_q (cm^{-1})	A	Γ (cm^{-1})	ω_q (cm^{-1})	A	Γ (cm^{-1})	ω_q (cm^{-1})	A	Γ (cm^{-1})
2900±1*	-1551±1*	390±1*	$\chi_{NR}^{(2)}$ = (-0.56±0.01)+i (-0.34±0.01)			-	-	-
2839±1	2.3±0.3	12±1	2840±2	0.1±0.1	10±2	2840±1	0.10±0.01	7±1
2885±1	2.0±0.6	15±1	2891±1	0.2±0.1	14±1	2875±1	0.17±0.01	8±1
2912±1	6.0±0.2	18±1	2912±1	0.5±0.1	12±1	2905±1	0.16±0.01	8±1
2934±2	2.3±0.2	17±3	2946±1	0.6±0.1	10±1	2935±1	0.22±0.01	9±1
2945±1	16.6±0.1	18±1	2987±1	0.3±0.1	15±1	2951±1	0.62±0.01	8±1
2989±1	9.1±0.9	14±1	-	-	-	2990±3	0.06±0.01	11±5
3016±2	0.3±0.2	20±4	-	-	-	3016±3	0.11±0.01	11±1

* Parameters used for fitting the exciton resonance.

Table S2. Fitting parameters for the time-resolved SF spectra at various time delays from the MoS_2/PMMA interface (for Fig. 4 in the main text)

ω_q (cm^{-1})	A	Γ (cm^{-1})	ω_q (cm^{-1})	A	Γ (cm^{-1})
	-4.8 ps			-0.8 ps	
2841±1	3.5±1.3	17±4	2840±1	3.4±1.3	18±4
2938±2	15.0±2.4	17±1	2937±2	15.7±2.4	19±1
2984±2	10.2±0.9	15±1	2984±2	11.0±0.9	17±1
	-0.7 ps			-0.6 ps	
2840±1	3.3±1.3	18±4	2840±1	3.7±1.3	20±4
2937±2	14.7±2.4	18±1	2937±2	14.8±2.4	19±1
2984±2	11.3±0.9	17±1	2984±2	11.2±0.9	18±1
	-0.5 ps			-0.4 ps	
2841±1	3.7±1.3	20±4	2841±1	3.2±1.3	20±4
2938±2	15.3±2.4	19±1	2939±2	14.4±2.4	19±1
2984±2	11.0±0.9	18±1	2984±2	11.1±0.9	19±1
	-0.3 ps			-0.25 ps	
2844±1	3.2±1.3	21±4	2842±1	3.2±1.3	20±4

ω_q (cm^{-1})	A	Γ (cm^{-1})	ω_q (cm^{-1})	A	Γ (cm^{-1})
2939±2	13.9±2.4	18±1	2940±2	13.6±2.4	18±1
2984±2	11.2±0.9	19±1	2984±2	11.3±0.9	19±1
	-0.2 ps			-0.15 ps	
2842±1	3.2±1.3	22±4	2843±1	3.5±1.3	21±4
2940±2	12.7±2.4	18±1	2941±2	11.8±2.4	18±1
2985±2	11.4±0.9	19±1	2986±2	11.5±0.9	19±1
	-0.1 ps			-0.05 ps	
2844±1	3.2±1.3	17±4	2843±1	5.0±1.3	24±4
2941±2	11.3±2.4	18±1	2941±2	11.0±2.4	18±1
2988±2	11.8±0.9	19±1	2988±2	11.5±0.9	20±1
	0 ps			0.05 ps	
2843±1	6.0±1.3	26±4	2843±1	6.0±1.3	28±4
2942±2	10.1±2.4	18±1	2943±2	7.3±2.4	16±1
2989±2	11.0±0.9	20±1	2990±2	8.9±0.9	19±1
	0.1 ps			0.15 ps	
2843±1	7.4±1.3	31±4	2843±1	6.5±1.3	30±4
2942±2	7.2±2.4	15±1	2942±2	8.3±2.4	15±1
2988±2	9.0±0.9	19±1	2988±2	9.0±0.9	18±1
	0.2 ps			0.25 ps	
2842±1	6.1±1.3	29±4	2841±1	6.7±1.3	30±4
2942±2	8.6±2.4	16±1	2942±2	8.6±2.4	16±1
2988±2	9.4±0.9	19±1	2987±2	9.5±0.9	18±1
	0.3 ps			0.35 ps	
2841±1	5.8±1.3	29±4	2841±1	6.2±1.3	30±4
2941±2	8.6±2.4	16±1	2941±2	8.7±2.4	16±1
2987±2	9.4±0.9	18±1	2987±2	9.3±0.9	18±1
	0.4 ps			0.45 ps	
2842±1	5.9±1.3	28±4	2842±1	6.0±1.3	29±4
2941±2	8.7±2.4	16±1	2941±2	8.7±2.4	16±1
2987±2	9.3±0.9	18±1	2987±2	9.4±0.9	18±1
	0.5 ps			0.6 ps	

ω_q (cm^{-1})	A	Γ (cm^{-1})	ω_q (cm^{-1})	A	Γ (cm^{-1})
2842±1	6.0±1.3	29±4	2842±1	6.2±1.3	29±4
2941±2	9.1±2.4	16±1	2940±2	9.2±2.4	16±1
2988±2	9.5±0.9	18±1	2987±2	9.5±0.9	18±1
	0.7 ps			0.8 ps	
2842±1	6.2±1.3	29±4	2842±1	6.2±1.3	28±4
2940±2	9.3±2.4	17±1	2940±2	9.6±2.4	17±1
2987±2	9.5±0.9	18±1	2987±2	9.6±0.9	18±1
	0.9 ps			1 ps	
2842±1	6.1±1.3	28±4	2842±1	6.6±1.3	29±4
2941±2	9.3±2.4	17±1	2941±2	9.9±2.4	17±1
2987±2	8.9±0.9	18±1	2988±2	9.5±0.9	18±1
	1.1 ps			1.2 ps	
2842±1	6.1±1.3	28±4	2842±1	6.2±1.3	29±4
2940±2	9.1±2.4	16±1	2941±2	9.3±2.4	16±1
2987±2	9.5±0.9	18±1	2987±2	9.5±0.9	18±1
	5.2 ps			10.2 ps	
2842±1	6.2±1.3	28±4	2841±1	6.2±1.3	29±4
2940±2	9.3±2.4	16±1	2939±2	10.4±2.4	16±1
2987±2	9.6±0.9	18±1	2987±2	9.3±0.9	17±1
	20.2 ps			30.2 ps	
2841±1	6.1±1.3	29±4	2840±1	5.7±1.3	27±4
2938±2	11.0±2.4	17±1	2938±2	11.3±2.4	17±1
2986±2	9.4±0.9	17±1	2986±2	9.4±0.9	17±1
	40.2 ps			50.2 ps	
2841±1	6.0±1.3	27±4	2841±1	6.3±1.3	28±4
2938±2	11.9±2.4	17±1	2938±2	12.0±2.4	17±1
2985±2	9.7±0.9	17±1	2985±2	9.7±0.9	17±1

II. Fresnel coefficients at different interfaces.

We used the modified matrix formalism to calculate Fresnel coefficients for nonlinear optical responses from thin film systems that takes into accounts of multiple reflection effects in Ref. 35, for both the PMMA/air (Interface 1 in Fig. S1a) and the buried PMMA/silica substrate (Interface 2 in Fig. S1a) interfaces. Refractive indices of different media used for Fresnel coefficient calculation are obtained from https://refractiveindex.info/. Here we neglect the influence of the atomically thin TMDC monolayers on linear optical spectrum.

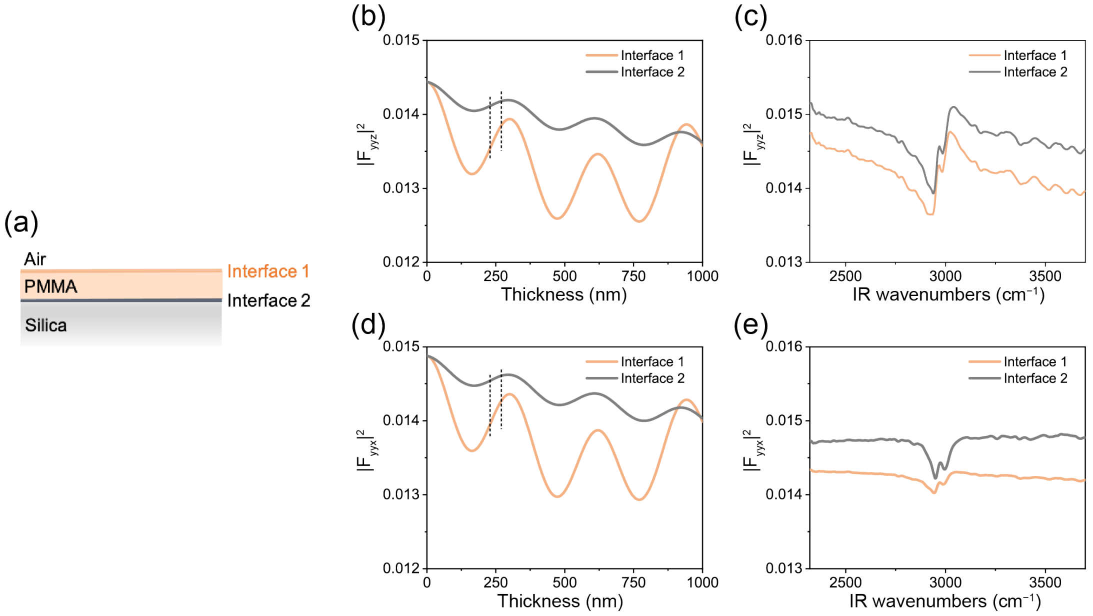

Figure S1. (**a**) Geometry of the air/PMMA/substrate system. (**b**) and (**d**) Calculated absolute square of total Fresnel coefficients $|F_{yyz}|^2$ and $|F_{yyx}|^2$ for both interfaces as functions of film thickness at ω_{IR} = 2900 cm^{-1}. (**c**) and (**e**) Calculated $|F_{yyz}|^2$ and $|F_{yyx}|^2$ for both interfaces as functions of IR wavenumbers for a PMMA film thickness of 250 nm.

III. Spectra from different sample spots.

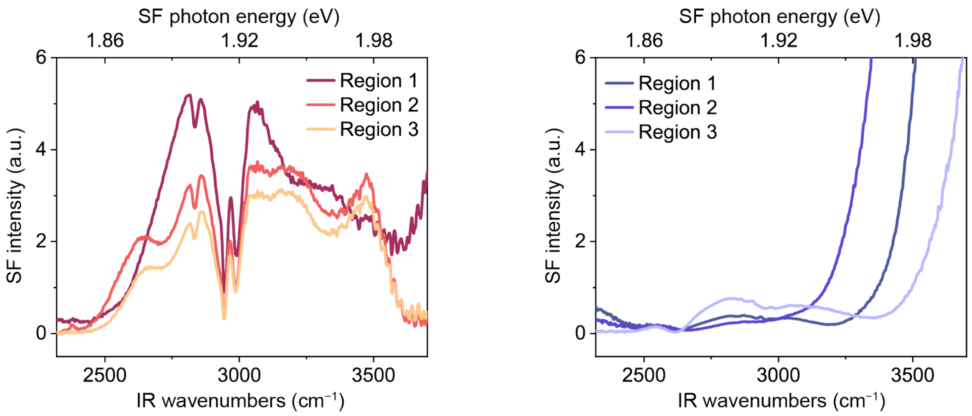

Figure S2. SF signals from the MoS_2/PMMA (left panel) and WS_2/PMMA (right panel) interfaces collected at different locations across the samples.

IV. The effect of TMDC sample size.

Experimentally, the diameter of the overlapped beam focus spots was ~ 420 μm, and the averaged lateral size of TMDC monolayers were ~200 and 300 μm. To estimate the effect of the TMDC sample size on the final result, we assume the portion of the area covered by TMDC is *r*, thus the total response from PMMA could be approximated by $\chi^{(2)}_{PMMA,tot} \propto r\chi^{(2)}_{TMDC/PMMA} + (1-r)\chi^{(2)}_{silica/PMMA}$. Here $\chi^{(2)}_{TMDC/PMMA}$ and $\chi^{(2)}_{silica/PMMA}$ denote the second-order nonlinear optical susceptibility of PMMA at the TMDC and silica interfaces, respectively. In the case of MoS_2 (upon excitonic resonance), we extracted the total response $\chi^{(2)}_{PMMA,tot}$ to be about 10 times greater than $\chi^{(2)}_{silica/PMMA}$, hence we have $\chi^{(2)}_{TMDC/PMMA} \sim [9/r+1]\chi^{(2)}_{silica/PMMA}$ (assuming the two $\chi^{(2)}_{PMMA}$'s have the same phase). Since $1 > r > 0$, this means $\chi^{(2)}_{TMDC/silica}$ could be even greater than 10 times of $\chi^{(2)}_{silica/PMMA}$. Using the beam spot diameter and sample size above, we have *r* about 25~60%, and we can estimate the enhancement factor to be another 1.6~3.7 times greater than the one extracted directly from the spectra.

V. Kinetic traces of the PMMA vibrational SF signal.

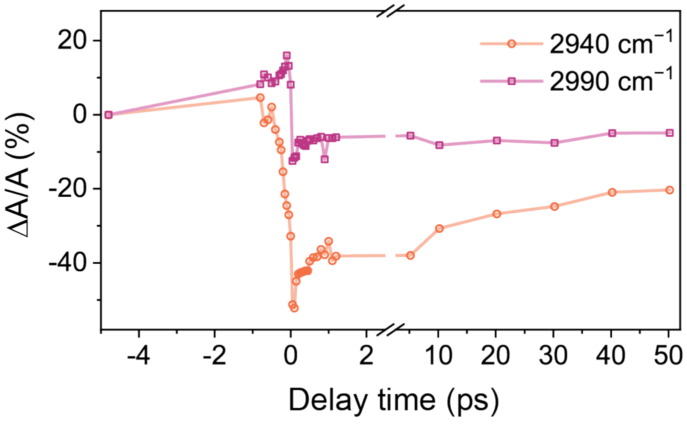

Figure S3. Kinetic traces of the PMMA vibrational SF signal at ~2940 and 2990 cm^{-1} at the MoS_2/PMMA interface.
